# Needs assessment for updating IOM standards for trustworthy clinical practice guidelines

**DOI:** 10.1002/gin2.70026

**Published:** 2025-05-26

**Authors:** Christopher Wolfkiel, Areeba Ahmed, Sandra Zelman Lewis, Mary Nix, Murad Alam

**Affiliations:** ^1^ Clinical Guidelines Resources Deerfield Illinois USA; ^2^ Department of Dermatology, Northwestern University Feinberg School of Medicine Chicago Illinois USA; ^3^ EBQ Consulting, LLC Santa Monica California USA; ^4^ Division of Practice Improvement, Center for Evidence and Practice Improvement Agency for Healthcare Research and Quality Rockville Maryland USA; ^5^ Department of Otolaryngology‐Head and Neck Surgery, Northwestern University Feinberg School of Medicine Chicago Illinois USA; ^6^ Department of Surgery, Northwestern University Feinberg School of Medicine Chicago Illinois USA

**Keywords:** clinical practice guidelines, IOM standards, trustworthy

## Abstract

**Background:**

The Institute of Medicine (IOM) Standards for Clinical Practice Guidelines We Can Trust (CPG Standards) and Standards for Systematic Reviews (SR Standards), established in 2011, have significantly influenced evidence‐based healthcare. However, the rapid evolution in medical practices and technologies necessitates a reassessment of these standards to ensure their continued relevance and effectiveness in modern healthcare.

**Methods:**

This study employed a survey approach targeting professional guideline developers. The first survey assessed the general need for updating IOM standards (both CPG and SR Standards), while the second focused on specific CPG Standards, soliciting detailed feedback on their current relevance and areas needing revision. Participants were purposively targeted from various medical specialties and roles in guideline development.

**Results:**

The General Need for Updating IOM Standards Survey garnered 22 responses, and the Specific CPG Standards Survey received 25 responses. A significant majority of respondents indicated the need for revising both the CPG and SR Standards. Key areas identified for CPG standards revision included conflict‐of‐interest management, incorporation of real‐world evidence and artificial intelligence, and systematic review processes. The responses highlighted the challenges of high compliance costs and the need for more practical execution guidance.

**Conclusion:**

This study highlights an urgent need for updating both sets of IOM Standards. The rapidly changing healthcare landscape, characterized by technological advancements and evolving medical evidence, necessitates a dynamic and responsive approach to guideline development. Establishing an authoritative body for periodic assessment and revision of these standards is crucial to ensure that CPGs remain scientifically robust, practical, and relevant to contemporary healthcare needs.

## Highlights

- This study was a survey of professional guideline development staff employed by guideline developing organizations in the United States who were challenged by the practicality of their organization's adherence to the IOM Standards.
- The study found that a significant majority of respondents believe that the 2011 IOM CPG Standards and the 2011 IOM SR Standards need updating.
- Key areas identified for revision in the CPG Standards include conflict‐of‐interest management, guideline development group composition (particularly patient involvement) and the intersection between guidelines and systematic reviews; SR Standards areas include incorporation of real world evidence and the impact of artificial intelligence.
- There was no clear consensus regarding which entity should be responsible for updating the standards, though a committee affiliated with the North American regional community of the Guidelines International Network was the leading choice (36.4%) for updating the CPG standards.

## 1 INTRODUCTION

As health‐related information grows and evolves, clinical practice guidelines (CPGs) are one of the linchpins that facilitate the delivery of evidence‐based, high‐quality care. Guidelines distill clinically relevant medical research into actionable, patient‐centered recommendations, helping clinicians and patients make informed decisions. As such, the credibility and reliability of guidelines is not just beneficial but essential for optimal patient outcomes especially in the highly specialized, multi‐payor regulated US healthcare environment. In 2011, the Institute of Medicine (IOM), now the National Academy of Medicine (NAM), at the direction of the Agency for Healthcare Research and Quality (AHRQ),^1^ undertook a seminal initiative to codify a dual set of standards aimed at underpinning the development of such trustworthy and methodologically rigorous guidelines.^2^ The CPG‐Standards were accompanied by systematic review development standards (SR Standards)^3^ (collectively referred to as IOM Standards). These were intended to serve as a model framework for US guideline developing organizations (GDOs) to develop their own methodologies for synthesizing the best available evidence and crafting actionable recommendations, while also providing a clear requirement for their regular updates and revisions.

More than a decade has passed since the IOM Standards were published, and the landscape of healthcare has undergone transformative changes. The rapid pace of medical innovation, the proliferation of digital health technologies, and the emergence of personalized medicine and the impact of environmental challenges have significantly altered the clinical horizon.^4^, ^5^ Pandemic‐fueled demands for rapidly developed and frequently updated guidelines also motivates guideline developers to adapt established methodologies. The CPG‐Standards and SR‐Standards of 2011 preceded today's evolving resource‐intensive challenges including implementation of computable guideline requirements^6^ and ever‐increasing new forms of evidence, such as real‐world data and AI‐analyzed outcomes (which were in their nascent stages when the standards were conceived^7^, ^8^). Moreover, the ethos of patient care has embraced a more collaborative model, wherein patient preferences and values are increasingly integral to the decision‐making process, a concept that was only beginning to gain traction a decade ago.^9^, ^10^

Interestingly, there are few evaluations in the literature of the continued relevance of the IOM Standards.^11^ While methodological advances in guidelines development by the GRADE working group^12^, ^13^ and others have continued apace, the IOM CPG and SR Standards have not continued to evolve despite the broader recommendations of the committees to build such infrastructure. These other methodological advances as well as other anecdotal evidence, suggest that the once‐groundbreaking IOM standards might no longer fully meet the evolving demands of modern medical practice or guideline development organizations.

Consequently, there is an imperative to scrutinize the IOM's CPG and SR standards through a contemporary lens, including operational perspectives. It is essential to discern which elements of these standards have withstood the test of time and continue to serve their purpose in guiding clinicians amidst the march of scientific progress, and which components have become obsolete or may require substantial modification to align with current and foreseeable healthcare principles and needs. An analysis of this type is not merely an academic exercise, but an urgent practical consideration, as the integrity of all CPGs directly impacts the quality of care delivered across the globe.^14^, ^15^

## 2 METHODOLOGY

To address the critical question of the relevance of the IOM Standards in the current healthcare landscape, this study surveyed professional guideline developers who are currently engaged in the development of CPGs. Purposive sampling was used to identify and query guidelines developers since the purpose of this needs assessment was to better understand how those using the IOM standards perceive their relevancy status relative to the pragmatic task of guideline development in the current healthcare context. The primary aim of this study was to capture a broad cross‐sectional range of opinions and perspectives within the community of US guideline developers.

Links to electronic surveys (Microsoft Forms) were sent by email beginning with the Specific CPG Standards Survey to approximately 50 professional guideline developers who participated in informal discussions as part of the Chicago Developers Group in June 2021 The emails included the option to forward to others in their organization who would be better suited to respond. The Specific CPG Standards Survey asked respondents to assess each of the 20 specific IOM CPG Standards, and determine which remained relevant, which may need to be reassessed in the future, and which needed to be reassessed now.

Repeated efforts to increase reach and response rate for the Specific Needs Survey were attempted including recruitment campaigns (email, meeting announcements, social postings etc.) directed at groups such as the Chicago Guidelines Developers group, Council of Medical Specialty Societies (CMSS) Guideline Developer Peer Group, Guidelines International Network – North America Regional Community (GIN‐NA), and relevant LinkedIn groups and authors' connections (*e.g*., Clinical Guideline Developers and the GIN Forum). Potential participants were informed that their responses were not required to be an official organizational position and that only one response from an organization affiliate would be allowed. As the response rate to the Specific Needs Survey plateaued the General Needs Survey was formed by removing the specific standards questions and directed at non‐responders. Finally, direct communications were made at the end of 2022 to significant non‐responding GDOs to respond to either survey.

The survey instruments were comprised of both open‐ended questions and multiple‐choice questions associated with Likert scales. This allowed for elicitation of both qualitative insights as well as the quantification of consensus on a range of issues, covering the entire spectrum of guideline development from evidence appraisal to recommendation formulation and dissemination. Both surveys contained general overview questions, while the Specific Needs Survey required respondents to choose for each of the 20 Trustworthy Standards whether it “Holds now and in the future”, “May need to be re‐assessed”, “Need to be re‐assessed now”, or “Other” and were averaged to assess the performance of the 8 “Proposed Standards” as defined by the IOM.^2^ The “Holds now and in the future” was considered the principal measure, as this metric best encompassed interpretation of the respondent's specific Standards feedback.

## 3 RESULTS

There were 22 responses to the General Need for Updating Survey and 25 to the Specific CPG Standards sSurvey. These respondents represented developers with more than 41 different organizational affiliations and 4 consultants with many GDO clients (Table 1). Respondents represented various medical specialties and disciplines and included a diverse array of professionals actively involved in guideline development, updating and implementation. They were academic researchers, those employed by professional societies or governmental bodies and other professionals integral to the guideline development process.

**Table 1:** Responder affiliations.

IOM CPG Survey: General Needs	IOM CPG Survey: Specific Standards
American Academy of Family Physicians	Academy of Nutrition and Dietetics
American Academy of Neurology	Academy of Orthopedic Physical Therapists
American Association for Respiratory Care	American Academy of Dermatology
American Cancer Society	American Academy of Family Physicians
American College of Gastroenterology	American Association of Sleep Medicine
American College of Medical Genetics and Genomics	American College of Chest Physicians
American College of Radiology	American College of Emergency Physicians
American College of Rheumatology	American College of Occupational and Environmental Medicine
American Gastroenterological Association	American Epilepsy Society
American Occupational Therapy Association	American Heart Association
American Psychological Association	American Society of Clinical Oncology
American Society for Parenteral and Enteral Nutrition	American Society of Gastroenterology Endoscopy
American Society for Radiation Oncology	American Society of Plastic Surgeons
American Society of Addiction Medicine	American Venous Foundations
American Society of Clinical Oncology	College of American Pathologists
American Thoracic Society	Congress of Neurological Surgeons
Emergency Nurses Association	Infectious Disease Society of America
Endocrine Society	North American Spine Society
Kaiser Permanente	Society of Critical Care Medicine (2)
Society for Vascular Surgery	Society of Thoracic Surgeons
Society of Interventional Radiology	Unaffiliated (4)
US Preventive Services Task Force	

### 3.1 General Need Survey and Specific CPG Survey general question results

Few respondents indicated that guideline (*n* = 2; 9.1%) and systematic review (*n* = 3; 13.6%) standards meet current and future needs (Table 2). Comments cited both the need for attention to the practicality of updated standards and for the inclusion of stakeholders in the updating process. Conflict‐of‐interest (COI) management processes were particularly concerning as one responder noted “More guidance on disclosure of interest and conflict of interest management including management of nonfinancial COI (aka intellectual COI), in general more guidance for how societies can more easily integrate these standards into practice in a practical way”. There was a lack of consensus regarding who should be responsible for updating the standards, yet another NAM committee was not the first choice of most responders for updating either systematic review or guideline standards. A consortium composed of systematic reviewers was the leading choice (*n* = 8; 36.4%) for who should update the Systematic Review Standards, and a committee affiliated with the North American regional community of the Guidelines International Network was the leading choice (*n* = 8; 36.4%) for updating the guideline standards. (Table 2).

**Table 2:** Who Should Update Systematic Review and Guidelines Standards, and How.

Needs Regarding Updating of Systematic Review Standards (*n* = 22)	*N* (%)
Most aspects are fine, but should be subject to review	11 (50%)
Some aspects need to be looked at now	7 (31.8%)
All standards meet current and future needs	3 (13.6%)
Other ‐ Overly prescriptive and detailed	1 (4.5%)

Needs Regarding Updating of Guideline Standards (*n* = 22)	
Most aspects are fine, but should be subject to review	11 (50%)
Some aspects need to be looked at now	8 (36.4%)
All standards meet current and future needs	2 (9.1%)
Other ‐ Aspirational	1 (4.5%)

Which Authority Should Update Systematic Review Standards (*n* = 22)	
Committee affiliated with GIN‐NA	6 (27.3%)
Another ad‐hoc NAM committee	5 (22.7%)
A new consortium of systematic review developers	8 (36.4%)
Committee affiliated with CMSS	1 (4.5%)
Other	2 (9.1%)

Which Authority Should Update Guideline Standards (*n* = 22)	
Committee affiliated with GIN‐NA	8 (36.4%)
Another ad‐hoc NAM committee	6 (27.3%)
A new consortium of guideline developers	4 (18.2%)
Committee affiliated with CMSS	2 (9.1%)
Other	2 (9.1%)

Comments on these standards indicated that they are perceived as too rigid and costly, with calls for more inclusivity of diverse evidence and practical considerations. Survey respondents questioned if higher trustworthiness standards enhance guidelines or merely inflate costs, with the implication that any updated standards should merge high ideals with real‐world practicalities and resources. Areas for updated standards consideration included adopting AI in systematic reviews, flexible evidence grading, and discussions between members of guideline and systematic review standard panels to allow alignment as proposed updated standards are assessed by different reviewing bodies. Regular updates were also deemed necessary to keep pace with medical advancements.

### 3.2 Specific CPG Standards Survey question results

**Figure 1:**
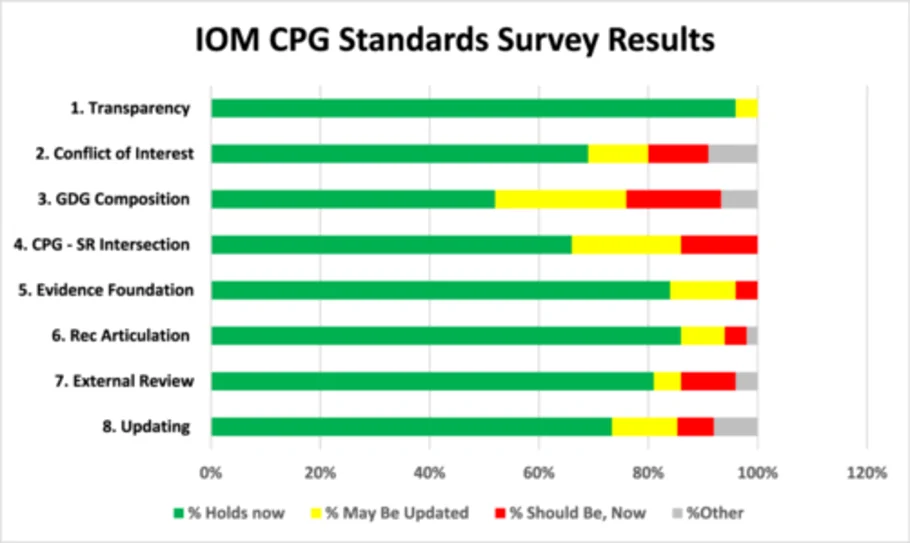
Percentage of Respondents Who Believe That IOM CPG Proposed Standards Holds Now and in the Future, May Need To Be Updated Now, or May Need To Be Updated In The Future.

The eight “proposed” CPG Standards results are graphed in Figure 1, based on the combined results of the 20 specific standards shown in Table 3. Proposed Standards “Holds now and in the future” (shortened to “Holds now” in the rest of this paper) ranged from 96% (Transparency) to 52% (GDG Composition). (Figure 1). Specific CPG standards pertaining to patient and public involvement (40%) and the increased involvement of other stakeholders (48%) had the lowest “Holds now” results. (Table 3). Specific CPG standards within the eight areas that respondents called for review with potential updates now or in the future included: systematic reviews, 56% “Holds now”, COI exclusions 64% “Holds now”, COI management 76% “Holds now” and financial divestment 64% “Holds now.”

**Table 3:** Specific IOM CPG Standard survey results (the number and percentage of respondents who responded “Holds now and in the future”, “May need to be re‐assessed now”, or “Needs to be re‐assessed in the future”).

Standards for developing trustworthy clinical practice guidelines	Holds now and in the future	Needs to be re‐assessed now	May need to be re‐assessed in the future	Other
1.1 Development transparency	24 (96%)	0 (0%)	1 (4%)	0 (0%)
2.1 Declarations of potential team members	18 (72%)	2 (8%)	3 (12%)	2 (8%)
2.2 Disclosures of COI with in CDG	19 (76%)	2 (8%)	3 (12%)	1 (4%)
2.3 Financial divestment	16 (64%)	3 (12%)	3 (12%)	3 (12%)
2.4 COI Exclusions	16 (64%)	4 (16%)	2 (8%)	3 (12%)
3.1 Multidisciplinary GDG	17 (68%)	1 (4%)	5 (20%)	2 (8%)
3.2 Patient and public involvement	10 (40%)	7 (28%)	6 (24%)	2 (8%)
3.3 Increased involvement strategies	12 (48%)	5 (20%)	7 (28%)	1 (4%)
4.1 CPGs based on IOM SR Standards	14 (56%)	6 (24%)	5 (20%)	0 (0%)
4.2 SR and CPG team interaction	19 (76%)	1 (4%)	5 ((20%)	0 (0%)
5.1 Recommendation requirements	21 (84%)	1 (4%)	3 (12%)	0 (0%)
6.1 Actionable articulation	20 ((80%)	1 (4%)	3 (12%)	1 (4%)
6.2 Strong recommendation compliance implications	23 (92%)	1 (4%)	1 (4%)	0 (0%)
7.1 Multistakeholder external reviews	19 (76%)	5 (20%)	0 (0%)	1 (4%)
7.2 Confidentiality of external reviewers	20 (80%)	2 (8%)	2 (8%)	1 (4%)
7.3 Tracking reviewer's comments	24 (96%)	1 (4%)	0 (0%)	0 (0%)
7.4 Public draft notice	18 (72%)	2 (8%)	3 (12%)	2 (8%)
8.1 SR, CPG date stamps	18 (72%)	2 (8%)	3 (12%)	2 (8%)
8.2 Evidence monitoring	18 (72%)	1 (4%)	4 (16%)	2 (8%)
8.3 Updating important recommendations	19 (76%)	2 (8%)	2 (8%)	2 (8%)

Specific CPG standards' that were deemed most to “Holds now and in the future” were development transparency (96%), tracking external reviewers' comments (96%), and the expectation of compliance with strong recommendations (92%). (Table 3).

The respondents in the Specific CPG Standards survey were asked whether there should be a similar follow up survey asking for feedback on the currency or relevance of each of the systematic review standards, much as the relevance of each individual guideline standard was queried in this survey. The vast majority (84%) of respondents agreed on the importance of this additional survey.

The Specific CPG Standards survey also asked respondents to consider the process for potential updating of these standards. The vast majority (88%) recommended routine assessments and updates to the IOM CPG Standards, with 60% suggesting a cyclical review every 5 years (Table 4). Almost half (48%) of respondents envisioned the stewardship of these standards under an independent non‐profit entity, albeit with certain contingencies concerning the choice of the organization as there was no clear consensus on which type of entity should serve as the steward for either the guideline or systematic review standards and there was not a consensus of whether rules should be considered for guidelines certification (Table 4).

**Table 4:** Respondents' Recommendations Regarding Updating IOM CPG Specific Standards, Including Timeframe.

**Should we only update those specific systematic review or guidelines standards that require updating? (*n*** = **25)**	
Yes	21 (84%)
No	4 (16%)

**Should systematic review and guidelines standards be assessed and updated routinely? (*n*** = **25)**	
Agree	22 (88%)
Disagree	1 (4%)
Unsure	1 (4%)
Other	1 (4%)

**How often should the systematic review and guidelines standards be updated? (*n*** = **25)**	
Every 5 years	15 (60%)
Every 10 years	1 (4%)
Every year	4 (16%)
Unsure	5 (20%)

**Should the systematic review and guideline standards be “owned” by an independent non‐profit organization? (*n*** = **25)**	
Agree	12 (48%)
Disagree	5 (20%)
Unsure	4 (16%)
Other	4 (16%)

**Should rules be established for guidelines certification? (*n*** = **25)**	
Agree	9 (36%)
Disagree	0 (0%)
Unsure	16 (64%)

## 4 DISCUSSION

The General Need respondents clearly indicated a necessity for updating both the CPG and SR IOM standards with only 9% agreeing that the standards “Holds now and in the future” (Table 2). While there was less of a consensus around which specific CPG standard areas needed updating (Figure 1), Conflict of Interest, Guideline Development Group Composition (especially 3.1 patient and public involvement Table 3) and Guideline‐Systematic Review Intersection were noted to be the specific CPG standards of most concern. This underscores the foundational aspects of guideline development, the people involved and the evidence they interpret, and the need for realistic standards that can be reliably met to insure trustworthiness. The importance of these issues in an era in which sources of medical information and funding are increasingly complex necessitates a robust and transparent approach. It is important that significant sources of financial and nonfinancial COI are not inadvertently excluded or obscured.^16^ Integration of additional stakeholders into the development process, particularly patients, has been identified by several responders as problematic and representative of potentially unrealistic standards. As one responder noted “Patient and consumer representation is vital. However, I speculate how vital it is to the entire guideline process ‐ do patients really need to be able to appraise the evidence? A patient's expertise is in their experience with the condition. There seems to be key points in the process this expertise is helpful, but appraisal of evidence is not particularly one of them. I believe there are ways to incorporate patient expertise that are more beneficial (question development, outcome discussions, implementation considerations, etc.).”

The integration of real‐world evidence (RWE) into the evidence base^17^ represents another significant shift for which systematic reviewers and guideline developers need to account. And, as one responder noted the use of AI machine learning in title and abstract screening has become increasingly utilized in the absence of standards as the productivity gain is undeniable. As the surveys were conducted before large language semantic models such as OpenAI, Anthropic and others became widely available, it is likely a similar productivity scenario will occur as they are introduced into the broader SR processes. The challenge here is threefold: First, to determine whether these newer forms of evidence and technologies should even be incorporated and how to best integrate them into existing systematic review and guideline development methods. Second, if the first challenge is met, to establish methodologies that can effectively synthesize and integrate such diverse forms of evidence and AI tools. Finally, to ensure that these methodologies are accessible and feasible for guideline‐developing organizations, many of which may face resource constraints. This shift towards embracing newer forms of evidence and technologies reflects the evolving landscape of healthcare, where data‐driven and patient‐centric approaches are increasingly respected. Clear and practical standards for managing conflicts of interest, forming development groups, and supporting evidence are essential for maintaining the trust of clinicians and patients in clinical practice guidelines.

While the respondents are representative of operational guideline development, they are part of a larger guideline ecosystem that includes executive leadership, members/volunteers and others such as publishers, implementation specialists, quality measure developers. Balancing requirements for developing trustworthy guidelines involves more than incorporating the Standards and those with budget/resource allocation responsibilities need to be aware of the overall timeline/quality balance that all developers face.

These surveys' results should be interpreted with the need for a more organized approach to updating guideline standards.^6^ In the face of rapid advancements in medical science, a static guideline document risks becoming outdated soon after its publication. Regular reviews and updates for living guideline processes were recommended by a significant proportion of respondents to ensure that the CPGs remain relevant and reflective of the latest clinical evidence and practices. One responder noted that an interpretation of IOM Standards as guiding principles requires less review and potential updating compared to operational checklists such as AGREE,^18^ RIGHT^19^ and GIN‐McMaster Guideline Development Checklist.^20^ Also noted was that the definition of “living guidelines” is needed to promote consistent representation to the market.

The lack of consensus on which entity should be responsible for overseeing updates to the IOM (NAM) Standards points to a broader issue of governance and stewardship in guideline development.^10^ While the IOM Trustworthiness authors also issued recommendations for US Agencies to address these issues, they have been largely ineffective and/or unheeded. Establishing a stakeholder agreed mechanism for review and update of US guideline standards, possibly under the aegis of a dedicated authoritative body, could provide the necessary oversight and ensure that guideline methodologies evolve in line with the changing needs of healthcare while staying true to the science and art of evidence‐based medicine.

In summary, the surveys' results not only highlight the standards in need of revision but also emphasize the broader context in which these processes operate. The discussion must pivot to contemplating the ways in which the medical community, guideline developers, and relevant authorities can collaboratively work toward standards and guidelines that are not only scientifically robust and trustworthy but also adaptable and responsive to ongoing changes in how healthcare is delivered, and evidence is generated in an evolving world.

## 5 LIMITATIONS

Limitations of this project include the relatively small number of respondents resulting from targeting individuals tasked with guideline development on the US and missing representation of significant GDO's (characterized as organizations with well resourced, high‐volume guideline production). We do estimate that this sample of 41 organizations is a representative sample of USGDO's with 75 identified by Guidelines Central (https://www.guidelinecentral.com/guidelines/) as having produced 2 or more guidelines over the last 2 years and note that these results may limit generalizability, especially with respect to the individual Standards. These results should not be attributed to any other stakeholder in the guideline ecosystem including dedicated systematic review experts, patients, providers or payers impacted by guidelines and others within the guideline developing organization including those with the authority to establish budgeting policy. As such, a more complete market survey may be required, and this study serves as a precursor of that potential effort.

These surveys prioritized single responses from guideline developers from those with operational responsibility since we believed that they had the most complete understanding of the development process. We were not looking for organizational responses, which we encourage as a follow‐up effort. Additionally, other stakeholders that may have valuable insight include patients, providers and payers impacted by guidelines produced under these standards. Also, systematic review leaders such as evidence‐based practice centers were not targeted – the intent was that their views would be addressed in a subsequent survey that addressed the specific standards of the SR standards.^3^

Multiple attempts were made to significant GDOs to respond to either survey. A broader response could be further enhanced through use of qualitative non‐survey collaboration models such as workshops, focus groups, and one‐on‐one interviews. And, as this effort was undertaken by individuals involved in guideline development, it should be viewed as market feedback that may need to be more rigorously verified with appropriate sponsorship and funding.

## 6 CONCLUSION

These survey results underscore the urgent need for a US guideline standards setting body to take responsibility for the periodic assessment and updating of the core standards for clinical practice guideline development. This standards‐setting body could also be responsible for assessing the impact of these standards in related tools such as quality measures and other related implementation efforts. This body's role would be crucial in ensuring that the process of CPG development stays abreast of evolving ethical, scientific, US healthcare and procedural expectations emerging from rapid advancements in medical science and technology. The body may, from time to time, incorporate the most generalizable research advances produced by guideline methodologists and developers. At the same time, the body should also be vigilant, collaborative, and proactive in involving a diverse array of stakeholders and partners in the healthcare community to review current standards for potential revisions. In summary, the IOM standards can only remain relevant if a guidelines standards setting body takes timely action to update those standards that require revision, while incorporating the views of key stakeholders, and committing to ongoing dedicated stewardship of guidelines standards. Convening a guidelines standards setting body should be the result of input from US stakeholders including GDOs (their academic thought leaders and professional staff), provider groups, payers such as CMS, and patient representatives.

## SPECIAL THANKS TO NON‐AUTHOR SURVEY RESPONDENTS

Abdul Abdullah, Deana M. Baptiste, Michael J Barry, Amy Bennett, Mary Bodach, Lisa Bradfield, Susan Cahill, Carol Colasacco, Maureen Corrigan, Altair Delao, Kate Einhaus, Scott Firestone, Lindsy Frazer‐Green, Lynda Goodfellow, Raquel Halfond, Jung Min Han, Lori Harmon, Join Heald, Kurt Hegmann, Jon Iaccarino, Joy Keller, David Kurth, Susan Lacey, Jennifer Malinowski, Deberah McBride, Christine McDonough, Liam McKeever, Laura Mitchel, Michael Monroe, Claire Neumann, Tom Oliver, Sheena Patel, Patricia Rehring, Craig Robbins, Travis Schultz, Neil Sengupta, Amy Skuteris, Sherene Thomas, Amy Turner, Melissa Weimer, Lauren Willhoit, Kevin C. Wilson, Lauren Zych.

## AUTHOR CONTRIBUTIONS

**Christopher Wolfkiel:** conceptualization, data curation, formal analysis, investigation, methodology, project administration, resources, supervision, validation, visualization, writing – original draft, writing – review and editing. **Areeba Ahmed:** resources, writing – review and editing. **Sandra Zelman Lewis:** conceptualization, investigation, methodology, resources, supervision, validation, visualization, writing – original draft, writing – review and editing. **Mary Nix:** investigation, supervision, writing – original draft, writing – review and editing. **Murad Alam:** investigation, resources, supervision, valodation, visualization, writing – original draft, writing – review and editing.

## CONFLICT OF INTEREST STATEMENT

The authors declare no conflicts of interest.

## ETHICS STATEMENT

Not applicable.

## Supporting information

CGD Supplementary.

IOM Standards Contributorship.

## Data Availability

The datasets generated during and/or analyzed during the current study are available from the corresponding author upon reasonable request.
