# Supplementary material for: Needs assessment for updating IOM standards for trustworthy clinical practice guidelines
Source: Clin Public Health Guidel. 2025 May 26;2(3):e70026. doi: 10.1002/gin2.70026 (PMC13563388; doi:10.1002/gin2.70026)
Supplement: Supplementary file 1 [file GIN2-2-e70026-s002.docx]

**Supplementary Figure 1: Need for Updating IOM (NAM) Trustworthy CPGs and Systematic Review Standards**

*Required

Next Steps for CPGs We Can Trust and Standards for Systematic Reviews

1. Please enter your name

……………………………

2. Please enter your email address

……………………………

3. Please enter your organization

……………………………

4. In general, how would you rate your organization's compliance with IOM/NAM Clinical Practice Guidelines We Can Trust standards? *

- Good
- Good, affirmed by ECRI
- Could be better
- Could be better, evidenced by ECRI
- Other

5. Overall, how would you rate IOM/NAM Clinical Practice Guidelines We Can Trust standards? *

- Critically outdated, needs immediate updating
- Some aspects need to be looked at now
- Most aspects are fine, but should be subject to a scheduled review
- All standards meet current and future needs
- Other

6. What would be your recommended authority to review/update Clinical Practice Guidelines standards?

- Another ad-hoc NAM committee
- Committee affiliated with GIN-NA
- Committee affiliated with CMSS
- A new consortium of guideline developers
- Other

7. Would your organization be willing to participate in an IOM/NAM Clinical Practice Guidelines "standard by standard" survey?

- Yes
- No
- Maybe
- Other

8. Do you have any comments on the need for updating IOM/NAM Clinical Practice Guidelines standards?

……………………………

9. In general, how would you rate your organization's compliance with IOM/NAM Standards for Systematic Reviews?

- n/a (we do not conduct SRs)
- Poor (few standards would be met)
- Fair (standards met comparable to peer organizations)
- Good (most standards met)
- Excellent (we continually assesses how well each project meets the standards)
- Other

10. Overall, how would you rate IOM/NAM Standards for Systematic Reviews?

- Critically outdated, needs immediate attention
- Some aspects need to be looked at now
- Most aspects are fine, but should be subject to review
- All standards meet current and future needs
- Other

11. What would be your recommended authority to review/update Standards for Systematic Reviews?

- Another ad-hoc NAM committee
- Committee affiliated with GIN-NA
- Committee affiliated with CMSS
- A new consortium of systematic review developers
- Other

12. Would your organization be willing to participate in a Standards for Systematic Reviews "standard by standard" applicability survey?

- Yes
- No
- Maybe
- Other

13. Do you have any comments on the need for updating Standards for Systematic Reviews?

……………………………

14. Do you have any additional comments regarding guidelines development or systematic review standards or compliance with them?

……………………………

15. Do you have any thoughts on dissemination or implementation potential standards such as those being constructed by the CPG-on-FHIR effort?

……………………………

**Supplementary Figure 2: Updating IOM/NAM Trustworthy Guideline Standards Survey**

In 2011 the IOM detailed two sets of criteria for modern guideline development in "Clinical Practice Guidelines We Can Trust" and "Finding What Works in Health Care Standards for Systematic Reviews". The purpose of this survey is to assess the currency of these Standards and the need for updating them.

**Clinical Practice Guidelines We Can Trust**

Each standard's recommendations have an option for you to opine on its currency and a free text "other", please select one of the options and add "other" feedback if desired. There is the option to add other recommendations or comments that should be considered in each section.

1. Please enter your email address

……………………

2. How long have you been involved with developing guidelines?
   - 0-5 yrs
   - 6-10 yrs
   - 10+ yrs

**a. Establishing Transparency**

3. The processes by which a CPG is developed and funded should be detailed explicitly and publicly accessible.
   - Standard holds now and in the future
   - Standard may need to be re-assessed for future applicability
   - Standard needs to be re-assessed for applicability now
   - Other
4. Other "Transparency" Standards Needed?

………………….

5. Comments on "Transparency" Standards

………………….

**b. Management of Conflict of Interest (COI)**

6. Prior to selection of the guideline development group (GDG), individuals being considered for membership should declare all interests and activities potentially resulting in COI with development group activity, by written disclosure to those convening the GDG:

Disclosure should reflect all current and planned commercial (including services from which a clinician derives a substantial proportion of income), noncommercial, intellectual, institutional, and patient–public activities pertinent to the potential scope of the CPG.

- Standard holds now and in the future
- Standard may need to be re-assessed for future applicability
- Standard needs to be re-assessed for applicability now
- Other

7. Disclosure of COIs within GDG:

• All COI of each GDG member should be reported and discussed by the prospective development group prior to the onset of his or her work.

• Each panel member should explain how his or her COI could influence the CPG development process or specific recommendations.

- Standard holds now and in the future
- Standard may need to be re-assessed for future applicability
- Standard needs to be re-assessed for applicability now
- Other

8. Divestment:

Members of the GDG should divest themselves of financial investments they or their family members have in, and not participate in marketing activities or advisory boards of, entities whose interests could be affected by CPG recommendations.

- Standard holds now and in the future
- Standard may need to be re-assessed for future applicability
- Standard needs to be re-assessed for applicability now
- Other

9. Exclusions:

• Whenever possible GDG members should not have COI.

• In some circumstances, a GDG may not be able to perform its work without members who have COIs, such as relevant clinical specialists who receive a substantial portion of their incomes from services pertinent to the CPG.

• Members with COIs should represent not more than a minority of the GDG.

• The chair or co-chairs should not be a person(s) with COI.

• Funders should have no role in CPG development *

- Standard holds now and in the future
- Standard may need to be re-assessed for future applicability
- Standard needs to be re-assessed for applicability now
- Other

10. Other Standards Needed on "Conflict of Interest (COI)"?

………………….

11. Comments on "Conflict of Interest (COI)" Standards

………………….

**c. Guideline Development Group Composition**

12. The GDG should be multidisciplinary and balanced, comprising a variety of methodological experts and clinicians, and populations expected to be affected by the CPG

- Standard holds now and in the future
- Standard may need to be re-assessed for future applicability
- Standard needs to be re-assessed for applicability now
- Other

13. Patient and public involvement should be facilitated by including (at least at the time of clinical

question formulation and draft CPG review) a current or former patient, and a patient advocate or patient/consumer organization representative in the GDG.

- Standard holds now and in the future
- Standard may need to be re-assessed for future applicability
- Standard needs to be re-assessed for applicability now
- Other

14. Strategies to increase effective participation of patient and consumer representatives, including training in appraisal of evidence, should be adopted by GDGs.

- Standard holds now and in the future
- Standard may need to be re-assessed for future applicability
- Standard needs to be re-assessed for applicability now
- Other

15. Other Standards needed on "Guideline Development Group Composition"?

………………….

16. Comments on "Guideline Development Group Composition"

………………….

**d. Clinical Practice Guideline – Systematic Review Intersection**

17. Clinical practice guideline developers should use systematic reviews that meet standards set by the Institute of Medicine’s Committee on Standards for Systematic Reviews of Comparative Effectiveness Research.

- Standard holds now and in the future
- Standard may need to be re-assessed for future applicability
- Standard needs to be re-assessed for applicability now
- Other

18. When systematic reviews are conducted specifically to inform particular guidelines, the GDG and systematic review team should interact regarding the scope, approach, and output of both processes.

- Standard holds now and in the future
- Standard may need to be re-assessed for future applicability
- Standard needs to be re-assessed for applicability now
- Other

19. Other Standards Needed on "Clinical Practice Guideline – Systematic Review Intersection"?

………………….

20. Comments on "Clinical Practice Guideline – Systematic Review Intersection"

………………….

**e. Establishing Evidence Foundations for and Rating Strength of Recommendations**

21. For each recommendation, the following should be

provided:

• An explanation of the reasoning underlying the recommendation, including

- a clear description of potential benefits and harms;

- a summary of relevant available evidence (and evidentiary gaps), description of the quality (including applicability), quantity (including completeness), and consistency of the aggregate available evidence;

- an explanation of the part played by values, opinion, theory, and clinical experience in deriving the recommendation.

• A rating of the level of confidence in (certainty regarding) the evidence underpinning the recommendation

• A rating of the strength of the recommendation in light of the preceding bullets

• A description and explanation of any differences of opinion regarding the recommendation

- Standard holds now and in the future
- Standard may need to be re-assessed for future applicability
- Standard needs to be re-assessed for applicability now
- Other

22. Other Standards needed on "Establishing Evidence Foundations for and Rating Strength of Recommendations"?

………………….

23. Comments on "Establishing Evidence Foundations for and Rating Strength of Recommendations"

………………….

**f. Articulation of Recommendations**

24. Recommendations should be articulated in a standardized form detailing precisely what the recommended action is, and under what circumstances it should be performed.

- Standard holds now and in the future
- Standard may need to be re-assessed for future applicability
- Standard needs to be re-assessed for applicability now
- Other

25. Strong recommendations should be worded so that compliance with the recommendation(s) can be evaluated.

- Standard holds now and in the future
- Standard may need to be re-assessed for future applicability
- Standard needs to be re-assessed for applicability now
- Other

26. Other Standards needed on "Articulation of Recommendations"?

………………….

27. Comments on "Articulation of Recommendations"

………………….

**g. External Review**

28. External reviewers should comprise a full spectrum of relevant stakeholders, including scientific and clinical experts, organizations (e.g., health care, specialty societies), agencies (e.g., federal government), patients, and representatives of the public.

- Standard holds now and in the future
- Standard may need to be re-assessed for future applicability
- Standard needs to be re-assessed for applicability now
- Other

29. The authorship of external reviews submitted by individuals and/or organizations should be kept confidential unless that protection has been waived by the reviewer(s).

- Standard holds now and in the future
- Standard may need to be re-assessed for future applicability
- Standard needs to be re-assessed for applicability now
- Other

30. The GDG should consider all external reviewer comments and keep a written record of the rationale for modifying or not modifying a CPG in response to reviewers’ comments.

- Standard holds now and in the future
- Standard may need to be re-assessed for future applicability
- Standard needs to be re-assessed for applicability now
- Other

31. A draft of the CPG at the external review stage or immediately following it (i.e., prior to the final draft) should be made available to the general public for comment. Reasonable notice of impending publication should be provided to interested public stakeholders.

- Standard holds now and in the future
- Standard may need to be re-assessed for future applicability
- Standard needs to be re-assessed for applicability now
- Other

32. Other standards needed on "External Review"?

………………….

33. Comments on "External Review"

………………….

**h. Updating**

34. The CPG publication date, date of pertinent systematic evidence review, and proposed date for future CPG review should be documented in the CPG.

- Standard holds now and in the future
- Standard may need to be re-assessed for future applicability
- Standard needs to be re-assessed for applicability now
- Other

35. Literature should be monitored regularly following CPG publication to identify the emergence of new, potentially relevant evidence and to evaluate the continued validity of the CPG.

- Standard holds now and in the future
- Standard may need to be re-assessed for future applicability
- Standard needs to be re-assessed for applicability now
- Other

36. CPGs should be updated when new evidence suggests the need for modification of clinically important recommendations. For example, a CPG should be updated if new evidence shows that a recommended intervention causes previously unknown substantial harm; that a new intervention is significantly superior to a previously recommended intervention from an efficacy or harms perspective; or that a recommendation can be applied to new populations.

- Standard holds now and in the future
- Standard may need to be re-assessed for future applicability
- Standard needs to be re-assessed for applicability now
- Other

37. Other standards needed on "Updating"?

………………….

38. Comments on "Updating"

………………….

**i. Other Considerations**

39. Should we ask about each IOM Systematic Review Standards recommendation as we have for the Trustworthy Guideline recommendations?

- Yes
- No

40. Comments on updating the Systematic Review Standards

- Agree
- Disagree
- Other

41. The Standards should be assessed and updated routinely *

- Never, they are perfect
- Every 3 yrs
- Every 5 yrs
- Every 10 yrs
- Stagger sections for annual partial updates
- Other

42. How often should the Standards be updated? *

- Agree
- Disagree
- Other

43. The Standards should be "owned" by an independent non-profit *

- Agree
- Disagree
- Other

44. Non-profit should establish rules for guideline certification that can be licensed to certifying authorities. *

………………….

45. Please share any other opinions

………………….
